# The extent of protective footwear use among school-age rural children at high risk for podoconiosis and socio-economic correlates: A household cross-sectional survey in Southern Ethiopia

**DOI:** 10.1371/journal.pntd.0009791

**Published:** 2021-10-04

**Authors:** Abebayehu Tora, Getnet Tadele, Gail Davey, Colleen M. McBride

**Affiliations:** 1 Department of Sociology, Wolaita Sodo University, Wolaita Sodo, Ethiopia; 2 Department of Sociology, Addis Ababa University, Addis Ababa, Ethiopia; 3 Brighton and Sussex Centre for Global Health Research, Brighton and Sussex Medical School, University of Sussex, Brighton, United Kingdom; 4 School of Public Health, Addis Ababa University, Addis Ababa, Ethiopia; 5 Rollins School of Public Health, Emory University, Atlanta, Georgia, United States of America; University of Colorado Denver University of Colorado Medicine, UNITED STATES

## Abstract

**Background:**

Podoconiosis is preventable if genetically susceptible people wear shoes starting from an early age and do so consistently. However, lack of routine use of footwear is one of the major risk factors for podoconiosis and several other foot-related Neglected Tropical Diseases (NTDs). This study is aimed at describing the extent of footwear use among school-age rural children susceptible to the disease and investigating associated socioeconomic factors.

**Methods:**

Cross sectional surveys were conducted in 330 randomly selected households in Wolaita zone, southern Ethiopia. A household head and a child aged between 9 and 15 years were recruited from each household. Household heads provided socioeconomic data while children were asked about their footwear ownership and footwear use.

**Results:**

Nearly half (49.5%) of the children reported either walking barefoot or wearing under-protective footwear in a range of situations. Girls, older children, those in higher school grades, who belonged to families with higher socio-economic status, and those who owned a larger number of pairs of footwear reported more protective use of footwear. The linear regression model constituting the adequacy of footwear ownership and interaction term (i.e. family socioeconomic status by adequacy of footwear ownership) variables explained 30% of variance in the protective use of footwear (AR^2^ = 0.307). The interaction effect of adequate ownership of footwear and family socioeconomic status consistently predicted the protective use of footwear among children (β = -0.175, p<0.01) though the main effect of adequacy of footwear ownership was stronger (β = 0.507, p<0.001).

**Conclusion:**

Increased adoption of protective footwear is needed to effectively prevent school-age children living in endemic areas from developing podoconiosis and other neglected tropical diseases. Interventions aimed to improve the protective footwear use should consider approaches that also increase the socio-economic capacity of families in podoconiosis endemic communities.

## Author summary

Podoconiosis is preventable if genetically susceptible people wear shoes starting from an early age and do so consistently. However, lack of routine use of footwear since early childhood has been observed to be a major covariate of vulnerability to podoconiosis. The present study assessed the extent of protective footwear use and associated family socioeconomic factors among school-age rural children at high risk of the disease. Household heads and their children (aged between 9 and 15 years) participated in household surveys. Nearly half (49.5%) of the children were found to have less-protective use of footwear, either walking barefoot or wearing under-protective footwear in a range of situations. Girls, older children, those in higher school grades, those from families with higher socio-economic status, and those who owned a larger number of pairs of footwear reported more protective use of footwear. The protective footwear use among children is likely to increase with increase in family socioeconomic status and number of pairs of different types of footwear owned by the children. Increased adoption of protective footwear is needed to prevent school-age children living in endemic areas from developing podoconiosis and other neglected tropical diseases. Interventions aimed to encourage ownership of multiple pairs of footwear should use approaches that also increase the socio-economic capacity of podoconiosis-affected families.

## Introduction

The health benefits of footwear have been well recognized in the context of neglected tropical diseases. Apart from providing protection and comfort to the feet, footwear reduces vulnerability to a range of foot-related diseases. Common examples of diseases associated with barefoot walking include Buruli ulcer, cutaneous larva migrans, tungiasis, hookworm infection, soil transmitted helminth infection, strongyloidiasis, and leptospirosis [1]. These diseases primarily affect children between 5 and 15 years particularly in low and middle-income countries [2,3]. Footwear is also a major behavioral covariate associated with the development of podoconiosis [4,5]. Unlike other foot-related diseases, the proportion of school-age children affected by podoconiosis is small [6], since prolonged barefoot contact with irritant mineral particles in the soil [7] is necessary for development of disease. However, genetic susceptibility increases the chances of development of podoconiosis [5,8], and evidence indicates the importance of the interaction between high genetic risk in developing the condition and inadequate use of footwear. Yet rural residents in endemic areas hold misconceptions about the interaction of genetic susceptibility, soil exposure, and footwear use in causing podoconiosis [9,10] that operate as barriers to optimum use of protective footwear [11–13].

A study conducted in Wolaita zone of Southern Ethiopia reported consistent (i.e., all-day everyday) use of footwear among preschool children in podoconiosis-affected households to be only 31% [11]. In another study on school children consistent use of footwear was reported to be 54% though adherent soil observed on children’s feet suggested that some footwear (e.g., open sandals) was less effective in preventing soil exposure [12].

Though these reports provide important evidence, biases may have led to under-ascertainment of inadequate footwear use. In one study [11] children’s footwear use was reported by the caregivers and may have been affected by social desirability bias. In the second study [12] children were assessed during school hours resulting in overestimation of footwear use as children in rural areas tend to wear footwear to school [13,14]. We suggest that estimates of footwear use and the degree of protectiveness conferred might differ if children were reporting the behavior themselves and information was collected among children regardless of school attendance.

Additionally, there is a dearth of evidence regarding the influence of socioeconomic factors on footwear use among school-age rural children highly susceptible to podoconiosis. Previous qualitative studies have reported the role of financial constraints in limiting parents’ ability to provide adequate number of pairs of footwear for their children [13,14]. Social epidemiological studies have long observed a close connection between socioeconomic factors and health behaviors [15]. According to Giddens, socioeconomic factors create social circumstances that can constrain or enable preventive behaviors [16]. With socio-economic advantage comes resources and access to opportunity structures that increase the life chances and expand disease prevention capabilities [17,18]. According to Glymour and colleagues, individuals are forced to behave in unhealthy ways due to socio-economic constraints [19].

Studies into podoconiosis also have recognized the roles of socioeconomic circumstances of families in the practice of preventive behaviors. Studies among rural people in podoconiosis- endemic communities have observed a positive association of family socioeconomic status with adequacy of number of pairs of footwear owned and footwear use behavior [13,14]. Due to their inability to afford more than one pair of shoes, parents had to insist their children go barefoot on certain occasions. However, the influence of socioeconomic status of families and adequacy of footwear ownership has not been adequately studied in relation to adoption of podoconiosis preventive behaviors among school-aged rural children. The measurement of socioeconomic status was based mainly on self-reported monthly income and perceived socio-economic wellbeing which may not indicate the actual situation of households [13,14,20,21]. Additionally, our knowledge of the role of socio-demographic characteristics such as gender, age, and education in footwear use behavior is also limited. Studies suggested that socio-demographic variables not only shape social context and day-to-day realities, but also help us understand the distribution of health behaviors across different segments of population [15] which might in turn facilitate prioritization of population segments for behavioral change programs [22,23]. Thus, we planned this study to evaluate the association between protective footwear use and socio-economic factors in order to identify possible traction points for interventions to encourage footwear use among rural school-aged children. We hypothesized that family socioeconomic status and adequacy of footwear ownership predict the likelihood of protective footwear use among school-aged rural children in Wolaita zone, southern Ethiopia.

## Materials and methods

### Ethics statement

Ethical approval was obtained from the ethics committees of the Armauer Hansen Research Institute (AHRI) (Project reg. No. P035/15) and the College of Health Sciences, Addis Ababa University (Protocol number 047/15/Ext). The Wolaita Zone Administrative Bureau gave written permission to work in the community. MFI allowed their outreach clinic site staff to help in the identification of study participants. Caregivers confirmed their permission for a child to participate in the study by signing or thumb-printing on the consent form. Children above age 12 expressed their assent verbally in the presence of their caregivers as a witness, to ensure the assent process was without any coercion. The use of verbal assent from children was approved by the ethics committees.

### Study setting and sampling technique

Cross-sectional surveys were conducted with children from households in Wolaita Zone, southern Ethiopia in March 2016. The study setting of this research has been described in an earlier publication [24]. Households affected by podoconiosis were the target population of the study. A single survey sample size estimation formula was used to determine the required sample size, i.e. $n=\frac{1.96^{2}p(1-p)(DEFF)}{d^{2}}$ [25], where n = total sample size required, p = population proportion (population parameter), d = desired level of absolute precision (alpha value), 1.96 = z-score, DEFF = design effect. The population proportion was determined based on 31% point prevalence of observed shoe-wearing among pre-school children in podoconiosis-affected families [11]. Using this proportion, a design effect (DEFF) of 1, and a Z-value 1.96, the total sample size determined for the study was 330 households. Recruitment was restricted to three rural communities (Damot Pulasa, Ofa and Boloso Sore) selected from the sixteen active outreach clinic sites of the Mossy Foot International (MFI) organization. The criteria for selecting these communities were a) longer MFI service years, and b) larger number of registered patients. A list of affected families was obtained from the MFI head office. The MFI distributed shoes to younger children in affected families until 2013/2014 in all *kebeles* (lowest administrative unit) in which outreach clinics were running. The outreach clinic staff identified affected families (households) eligible for the study using the last shoe distribution list that included information about children’s age and sex, the number of siblings in the family who received shoes, and *kebele* of residence. The list of households in the shoe distribution list served as a sampling frame. The total number of podoconiosis patients registered by MFI in the area at the time of the study is larger than this, as shoes were only distributed to households with school-age children.

The sampling frame was composed of a total of 261 households in Damot Pulasa district, 405 households in Ofa district and 297 households in Bolo Sore district. Due to the variation in number of households by district, a probability proportionate to size sampling technique was used to select 90 households from Damot Pulasa district, 139 households from Ofa district and 101 households from Boloso Sore district. We kept twenty percent of sample households in the sampling frame of each district in reserve list so that data collectors could easily replace if an eligible respondent was absent. A list of sampled households coded with random numbers was given to each data collector. In every sampled household, a household head and a child were recruited. In households with more than one child in the age range, data collectors used a lottery method to select one child to complete the survey. The eligibility criteria for children were a) being 9 to 15 years of age, and b) being free from podoconiosis. Household heads provided socio-economic data for their family.

### Development of measures

Composite indexes were used to measure footwear use, ownership of footwear and family socio-economic status variables. Indices are composite measures that summarize and rank several indicators to represent the general dimension of a given concept. The indicators used to form an index may not necessarily be related to each other and the inter-correlation of items within the index is not a prerequisite to combining them [26]. A multidisciplinary panel of experts with many years’ experience of research on podoconiosis and other NTDs validated the content, relevance and clarity of the items in indices.

### Footwear use index

We conceptualized footwear use as a function of both *frequency* of footwear use across situations and *protectiveness* of footwear used. Frequency was indicated by the use of footwear in a range of situations as recalled over the last seven days, and as observed by the data collector on the day of interview. The index constituted 13 indicators probing the use of footwear in a range of situations. The situations include looking after cattle, farming, fetching water from the river, collecting fuel wood, walking around the homestead, playing games or sports exercises and social occasions such as going to market, church or school. These indicators have been reported in previous qualitative and quantitative studies as important situations in which the use of footwear varied [11–13]. Protectiveness, on the other hand, was indicated by features of the footwear used [20,27]. Closed footwear in which the footwear covered the entire foot was considered protective while open footwear (such as sandals) where areas of the feet are exposed was considered under-protective. For the purpose of computing total scores of the index, weights were given to the types of footwear included in a response scale format: 0 for None (if the respondent was not wearing any shoes), 1 for open (sandal shoes of any type), and 2 for closed (boots or closed shoes of any type).

### Adequacy of footwear ownership index

Adequacy of footwear ownership was defined as the number of pairs of footwear (of any type) owned by the school-aged child. Previous research has identified five general types of footwear including: open plastic/foam, closed plastic/foam, open leather, closed leather, and closed canvas/sneaker were included in the index as possible types of footwear owned [28–30]. The number of pairs of footwear a school-aged child owned was used to form a response scale and given a weight based on the opportunities to avoid barefoot exposure with increase in the number of pairs of footwear available: none = 0, one pair = 1, and ≥ two pairs = 2.

### Family socioeconomic status index

The Family Socioeconomic Status index was developed to measure multidimensional aspects of household level socioeconomic inequality among families affected by podoconiosis. Three dimensions (education, income, and occupation) have been widely used in the measurement of socioeconomic status [31,32]. However, these dimensions have low variability in developing countries [33]. As a result, an assets-based approach has been suggested for measuring the socioeconomic circumstances in these contexts [33,34]. Assets-based approaches are extensions of the sustainable livelihood framework (SLF) that focuses on specific household assets to determine socioeconomic position among families in the rural setting [34]. According to Kollmair and Gamper, “…people require a range of assets to achieve their self-defined goals as no single capital endowment is sufficient to yield the desired outcomes on its own” [35]. Thus, we conceptualized family socio-economic status as a function of ownership of important livelihood assets that include human assets (knowledge, skills and health), social assets (social networks and membership in groups), natural assets (natural resource stocks such as land), financial assets (cash and bank deposits) and physical assets (such as secure shelter). Indicators of assets included in the socio-economic status index were drawn from SLF and existing livelihood studies in Wolaita Zone and elsewhere in Ethiopia [34,36,37,38]. A total of 51 asset indicators were included in the family socioeconomic status index, and all required a dichotomous response, 0 for “No” and 1 for “Yes”. The wealth index scores were determined for family socio-economic status index through Principal Component Analysis (PCA). Excluding those asset indicators either owned by all (above 95%) or few (below 5%), a total of 35 asset indicators were included in PCA.

### Data collection

Experienced data collectors certified with a college diploma or degree in a social or health sciences field were recruited and trained. The trained data collectors pretested the instruments in six households in the study area to ensure understandability, clarity and relevance of the questions. Two data collectors were deployed per study site: one surveyed a parent household head while the other surveyed the child. The data collection was conducted using a pretested instrument in a location that provided privacy for the respondent. Data collection was supervised daily by three assistant supervisors and every three days by the first author. In each study site, the MFI outreach clinic staff collaborated with the *kebele* network leaders and other knowledgeable individuals in the village to link the sampled households with data collectors.

### Data analysis

A trained data entry clerk entered the data into a SPSS spreadsheet as coded in the questionnaire. Statistical Package for Social Sciences version #20 (SPSS), was used for the analysis.

Descriptive analysis was conducted using frequency distributions to describe socioeconomic status of households, adequacy of footwear ownership and extent of protective use of footwear among respondents. The independent samples T-test was employed to determine if the mean scores of protective use of footwear varied by gender (categorical), age (originally measured as interval variable and categorized into two groups using mean age as a cut-off point), or educational attainment (originally measured as interval variable and categorized into two using median school grade as a cut-off point). Pearson product-moment correlation analysis was conducted to determine the association between socio-economic factors such as family socioeconomic status (standardized scores) and adequacy of footwear ownership (standardized scores). A linear regression analysis was conducted to test whether socioeconomic factors such as family socio-economic status (standardized scores) or adequacy of footwear ownership (standardized scores) had independent or interaction effect on the protective use of footwear (standardized scores). To determine the interaction effect, a new product term variable was created, family socioeconomic status variable multiplied by adequacy of footwear ownership. The effects of gender (categorical), age (categorical) and educational attainment (categorical) variables were controlled. All variables were entered into the linear regression model using a backward stepwise method. Collinearity between variables was checked through a correlation matrix, in which none of the independent variables were strongly correlated. An alpha value of 0.05 was considered to determine the statistical significance of associations. A list-wise deletion method was used to exclude cases with missing values during computation of index scores and test of associations.

## Results

### Demographic characteristics of respondent children

As indicated in Table 1, 46.4% of the respondents were girls. The mean age of the respondents was 12.3 years (±1.89, range: 9–15). The educational attainment of school-aged children was measured by the number of school grades attained. Only 24 children (7.3%) had never attended school. The median school grade for those respondents who attended school was 3 (School grade range 1–10).

**Table 1:** Demographic characteristics of respondents (N = 330).

Variable	Category	n (%)	Mean (SD)	Median	Min (Max)
**Gender**	Boys	177 (53.6)	**-**	**-**	**-**
	Girls	153 (46.4)			
**Age**	-	-	12.3 (±1.89)	12	9 (15)
**Educational attainment**	Never enrolled	24 (7.3)			
	Enrolled	306 (92.7)	-	Grade 3	Grade 1 (Grade 10)

### Family socio-economic status of respondents

**Fig 1:**
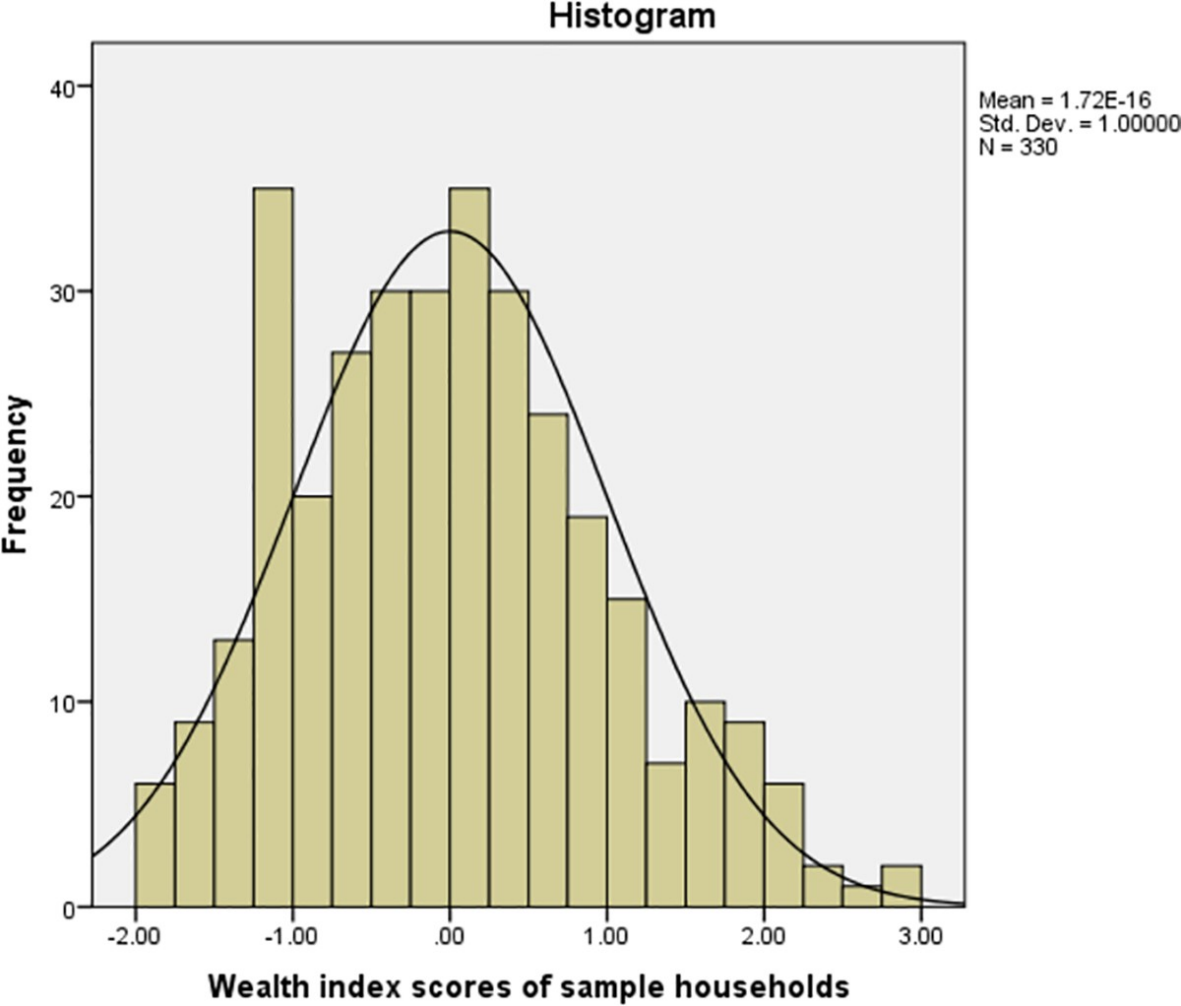
Wealth index scores indicating socioeconomic status of sample households.

As Fig 1 shows, wealth index scores of socioeconomic status of households ranged from -1.91 to 2.90, while zero represented an average wealth index score. Positive wealth index scores indicated better-off socioeconomic status households while negative wealth index scores indicated worse-off socioeconomic status households.

For descriptive purpose, we considered standard normal distribution of wealth index scores to classify households into five wealth quantiles: -2 to -1 = lowest, -1 to 0 = lower, 0 to 1 = middle, 1, 1 to 2 higher and 2 to 3 = highest. Households within 1 standard deviation were categorized into each category. Accordingly, as indicated in Table 2, the lowest socioeconomic status households accounted for 19.1% while the highest socioeconomic status households accounted for only 3.3%.

**Table 2:** Description of households by socioeconomic status (SES) categories (N = 330).

Variable	Category	N (%)	Wealth index scores		
			Mean (SD)	Median	Min (Max)
**Family SES**	Lowest	63 (19.1)	0.000 (±1.000)	-0.0408948	-1.91313 (2.90299)
	Lower	107 (32.4)			
	Middle	108 (32.7)			
	High	41 (12.4)			
	Highest	11 (3.3)			

### Adequacy of footwear ownership among respondents

Adequacy of footwear ownership was measured by an index of types and number of pairs of footwear owned by children. Table 3 presents the most commonly owned type of shoe, open plastic/foam (70.4%), followed by closed plastic/foam (41.8%), closed canvas (19.4%), closed leather (17.5%) and open leather (11%).

**Table 3:** Types and number of pairs of footwear owned by respondents (N = 330).

Types of footwear	Number of pairs of footwear owned		
	None N (%)	One pair N (%)	≥Two pairs N (%)
**Open-toed plastic/foam**	97 (29.4)	175 (53)	58 (17.5)
**Open-toed leather**	294 (89.1)	27 (8.2)	9 (2.7)
**Close-toed plastic/foam**	192 (58.2)	115 (34.8)	23 (7.0)
**Close-toed leather**	272 (82.4)	49 (14.8)	9 (2.7)
**Close-toed canvas/sneaker**	266 (80.6)	60 (18.2)	4 (1.2)

To determine the adequacy of footwear ownership, weights were given to the number of pairs of each type of footwear children reported owning (0 = none, 1 = one pair, 2 = ≥ two pairs). As indicated in Table 4, the total adequacy of footwear ownership index scores ranged from 0 to 9 (Mean = 1.96, SD = 1.37). For descriptive purpose, the mean score was used as a cutoff point, and the adequacy of footwear ownership was below average for 41.5% respondents.

**Table 4:** Adequacy of footwear ownership by respondents (N = 330).

Variable	Category	N (%)	Total footwear ownership scores		
			Mean (SD)	Median	Min (Max)
**Adequacy of footwear ownership**	Inadequate	137 (41.5)	1.96 (1.37)	2.00	0 (9)
	Adequate	193 (58.5)			

### Pattern of footwear use among respondents across situations

Patterns of footwear use varied across situations and activities (Table 5). Going barefoot was relatively common when children performed domestic chores at home (24.8%), were at home in their spare time (39.4%), engaged in farming (42.1%), or playing sports in school (36.7%) or outside school (36.7%). Closed footwear was used in very specific situations like going to church (50.3%), market (48.5%) or school (42.7%).

**Table 5:** Situation specific pattern of footwear use among respondents (N = 330).

Conditions	Types of footwear							
	Does not apply		None (without any footwear)		Open footwear		Closed footwear	
	N	%	N	%	N	%	N	%
**When performing domestic chores**	-	-	82	24.8	180	54.5	62	18.8
**When performing farming activities**	33	10	115	34.5	113	34.6	69	20.9
**While cutting grass**	16	4.8	48	14.5	182	55.2	84	25.5
**When collecting fuel wood**	8	2.4	43	13.0	177	53.6	102	30.9
**When looking after cattle**	27	8.2	35	10.6	179	54.2	89	27
**When fetching water**	4	1.2	37	11.2	202	61.2	87	24.4
**When playing games at school**	35	10.6	105	31.8	80	24.2	110	33.3
**When playing games after school**	32	9.7	121	36.7	113	34.1	64	19.4
**Walking around homestead (as observed at the time of interview)**	-	-	130	39.4	154	46.7	46	13.9
**When going to nearby market in the village**	11	3.3	28	8.5	149	45.2	142	43
**When going to big market in the town**	21	6.4	26	7.9	123	37.3	160	48.5
**When going to school**	31	9.4	24	7.3	134	40.6	141	42.7
**When going to church**	1	0.3	26	7.9	138	41.8	166	50.3

### Extent of protective use of footwear among respondents

All 13 items representing various situations of footwear use were considered to determine the total scores for the footwear use index. As shown in Table 6, the responses of the 220 cases who responded to all of the items in the index were summed to determine the total index scores. Respondents who responded “does not apply” for some items in the index were not considered during determination of total index scores. The total index scores were determined by summing the values in each situation (0 for barefoot, 1 for open footwear and 2 for closed footwear), and ranged from 0 to 26. The average score for footwear use was 14.3 with a standard deviation of 6.56.

**Table 6:** Extent of protective footwear use among respondents (N = 220).

Variable	Category	N (%)	Footwear use scores		
			Mean (SD)	Median	Min (Max)
**Footwear use**	Less protective	109 (49.5)	14.3 (±6.56)	15.0	0 (26)
	More protective	111 (50.5)			

### Association of demographic factors with extent of protective use of footwear among respondents

As shown in Table 7, the independent samples T-test was used to determine the association of protective use of footwear with gender, age and educational attainment of respondents. Girls were found to be more likely to have protective use of footwear than boys (mean = 15.6, standard deviation (SD) 6.3, vs 13.42, SD 6.6), and this difference was statistically significant (t = -2.441, df = 218, p = 0.015). The age and educational attainment variables originally measured at interval scale were transformed into categories using the median as a cut-off point. Respondents in early adolescence (13–15 years) reported more use of protective footwear (mean = 15.21, SD = 4.81) compared to respondents of 9–12 years (mean = 13.26, SD = 7.84), and the difference was statistically significant (t = - 2.318, df = 214, p = 0.021). Similarly, a statistically significant difference was observed in the use of footwear between respondents who had attained below grade 4 than above (mean = 12.60, SD = 7.39 vs. 16.06, SD = 5.02, t = - 4.054, df = 218, p <0.001).

**Table 7:** Association of protective footwear use with demographic characteristics of respondents (N = 220).

Variable	Category	N	Mean (SD)	Mean difference		
				t-value	df	P-value
Gender	Boys	131	13.42 (±6.61)	- 2.441	218	0.015
	Girls	89	15.6 (±6.30)			
Age	Middle childhood (9–12)	107	13.18 (±7.86)	- 2.499	218	0.013
	Early adolescence (13–15)	113	15.36 (±4.84)			
Educational attainment	≤ Grade 3	112	12.60 (±7.39)	- 4.054	218	<0.001
	≥ Grade 4	108	16.06 (±5.02)			

### Association of socioeconomic factors with protective use of footwear among respondents

As indicated in Table 8, the Pearson correlation coefficient revealed a positive association between protective use of footwear with family socioeconomic status (r = 0.204, p<0.01) and adequacy of footwear ownership (r = 0.526, p<0.01).

**Table 8:** Association of protective footwear use with socioeconomic (SES) factors.

Variables	Correlation	Regression model without interaction term variable		Linear regression models with interaction term variable							
	r (p value)	β (p value)	AR^2^	Model 1		Model 2		Model 3		Model 4	
				β (P-value)	AR^2^	β (P-value)	AR^2^	β (P-value)	AR^2^	β (P-value)	AR^2^
**Family SES**	0.204 (0.002)	0.093 (0.118)	0.278	0.103 (0.078)	0.305	0.103 (0.078)	0.308	0.101 (0.083)	0.310	0.110 (0.057)	0.307
**Adequacy of footwear ownership**	0.526 (<0.001)	0.467 (<0.001)		0.473 (<0.001)		0.472 (<0.001)		0.480 (<0.001)		0.507 (<0.001)	
**Family SES by Adequacy of footwear ownership**	-	-		-0.172 (0.003)		-0.172 (0.003)		-0.175 (0.002)		-0.175 (0.002)	

**Fig 2:**
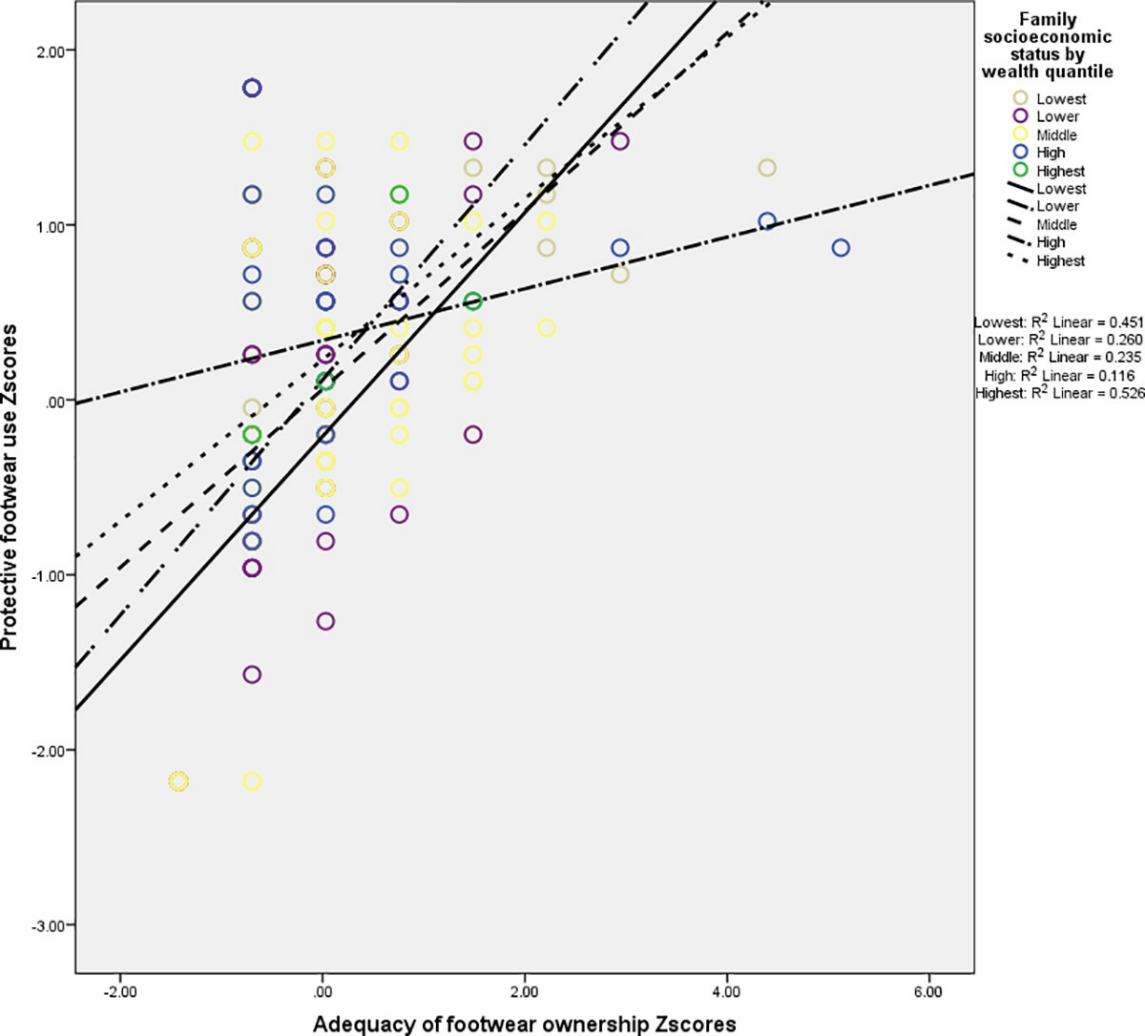
The interaction effect of socioeconomic factors on protective footwear use among respondents.

The exploratory regression analysis revealed the effects of family socioeconomic status and adequacy of footwear ownership on protective use of footwear when demographic attributes of respondents such as age (categorical), sex (categorical) and educational attainment (categorical) were controlled in all steps of the regression analysis. Regression analysis stopped at the fourth step. The final model, in which family socioeconomic status, adequacy of footwear ownership and product term variables remained, explained 31% of variance in the protective use footwear among respondents. The main effect of adequacy of footwear ownership on protective use of footwear remained consistent (β = 0.507, p<0.001) while the main effect of family socioeconomic status was not consistent. Interestingly, the interaction between family socioeconomic status and adequacy of footwear ownership was statistically significant (β = -0.175, p<0.01). Fig 2 shows the interaction between family socioeconomic status and adequacy of footwear ownership footwear in influencing the protective use of footwear among school-aged children.

## Discussion

This study aimed to measure the extent of protective footwear use among school-age rural children in Wolaita zone using a composite index of context-driven indicators. Efforts were made to systematically measure concepts such as socioeconomic status and adequacy of footwear ownership and to investigate their association with the protective use of footwear. Based on the wealth index scores, the proportion of households falling into the lowest socioeconomic status categories was 19%, while only 3.3% of the households belonged to the highest socioeconomic status. This may not be surprising as podoconiosis-affected families are known to face a double burden, being prevented from economic and social activities and impacted by the costs of treatment [39]. Studies have also indicated the debilitating impacts of podoconiosis-related stigma on the socio-economic wellbeing of affected individuals and their families [40,41].

Almost all children participating in this study reported ownership of some form of footwear. This demonstrates a positive change in the lifestyle of rural people in Ethiopia, where a barefoot tradition has prevailed for a long period of time. Nevertheless, the types and numbers of pairs of footwear owned by rural children are very limited, with only two pairs owned on average. The most common types of footwear owned by children were open plastic shoes in contrast to adult members of the rural population in settings known for podoconiosis endemicity in Ethiopia. For example, a study reported ownership of open plastic footwear among only 18% of adults affected by podoconiosis and 29% of adults never affected by podoconiosis [20]. On the other hand, the ownership of closed footwear of any type was lower for children in this study compared to adults. In a previous study, around 60% of adults reported owning closed plastic, leather and canvas types of footwear [20]. This suggests that adults are more privileged than children in terms of access to more pairs of protective footwear.

Our assessment of protective footwear use among school-aged rural children provided important evidence on the vulnerability of rural children in the study area to podoconiosis. A range of previous studies have described footwear use in podoconiosis-endemic communities [20,27,29,30], but most have relied on self-reported information or age at first use of footwear and footwear use at a single point in time (at interview), which make it difficult to assess whether footwear is used consistently and optimally across situations in daily life. A few studies attempted to measure consistent use of protective footwear and observed it to be inadequate [12,42]. This study also further enhances our understanding of patterns of shoe use across a range of situations, and will enable more flexible, targeted responses (e.g. footwear aimed at specific use scenarios). In the present study, the proportion of children going barefoot in situations like domestic chores, farming and sports activity ranged from 25% to 40%. This is consistent with previous studies [13,14]. Barefoot exposure among children performing various activities was found to be higher than that among adults. In Alemu and colleagues’ study [29], 22% of adults reported walking barefoot when farming and 11% reported walking barefoot at home. Similarly, 17% of adults reported going barefoot during farming, 16% during other work and 30% at home in Molla and colleagues study [28]. In another similar study, only 17% of adults reported walking barefoot during farming, while 13% indicated that they went barefoot at home [30]. As is the case for adults [13,14], children’s use of footwear at social occasions was found to be high.

Overall in this study, the extent of protective footwear use was low among school-age rural children in Wolaita zone. Nearly half (49.5%) of the children recruited from podoconiosis-affected families were found to use footwear in a way unlikely to afford them protection from the soil. This is congruent with Watanabe and colleagues’ study [12], that reported inconsistent footwear use in 46% of children recruited in a rural school setting. However, the proportion of school-age children with inadequate footwear use in this study was lower than that of preschool children in a previous study [11]. This may partly be attributed to the age difference between the studies, making preschool children more disadvantaged than school-age children.

The association between gender and protective footwear use is another important finding. The protective use of footwear among girls was found to be better than used footwear more protectively than boys. This may be baffling considering the deeply entrenched privilege of men that stems from cultural and institutional bias against women in societies, particularly in low and middle income settings [43–45]. However, studies have often shown that women have healthier lifestyles: they eat healthier foods, drink less alcohol, smoke less, and use seat belts more frequently compared to men who outperform women only in physical activity [15]. The gender and hygiene hypothesis highlights the tendency of girls to maintain hygiene better than boys and that girls are more often dressed in clothing that is not supposed to get dirty and receive more parental supervision and direction regarding cleanliness than do boys [46]. In the context of podoconiosis, studies have reported inconsistent results on differences in footwear use behavior between boys and girls. Watanabe and colleagues [12] reported that girls were more consistent in using shoes than boys. In contrast, parents reported higher levels of shoe wearing among preschool-age boys than girls in Ayode and colleagues’ study [11]. These inconsistencies indicate the need for more efforts to the gender dynamics in protective use of footwear among school-aged rural children. The privileged status of girls in the protective of use of footwear may be related to cultural or economic factors which need further investigation.

Protective footwear use tended to increase with children’s age and educational attainment–with age and progress to higher school grades, children are more likely to use more protective footwear regularly. This may be related to the parents’ tendency to provide footwear to children at the age they enroll in school [13]. As age increases children also start generating income by themselves through petty trade and other means which allows them to own shoes. However, the poor use of footwear in early childhood is likely to limit the probability of all-day-everyday use of footwear in adulthood as habits developed in childhood as predictive of habits in adulthood [47,48]. The inconsistency of footwear use among 50% of school-age children in this study and similar findings in previous studies [11,12] confirms the need of promotion of footwear use from early childhood.

A positive association between family socio-economic status and protective use of footwear among school-age rural children suggests the need for interventions aimed to improve socioeconomic wellbeing of podoconiosis affected families. Family socio-economic status alone does not guarantee regular use of footwear—having adequate resources alone will not ensure optimal preventive actions against podoconiosis. Interventions aimed to address misconceptions and increase motivation to wear shoes are also needed. Health promotion research in behaviors such as physical activity showed that poorer households prioritize available resources for other purposes [49,50]. This may be due in part to poor parents using less health promoting parenting approaches with their children as a means of coping with the stressful impacts of socio-economic difficulties [51].

A statistically significant association was also observed between adequacy of footwear ownership and protective use of footwear. Children owning few alternative pairs of footwear had less protective use of footwear. This is consistent with previous qualitative studies that reported shortage of alternative pairs of footwear to be an important determinant of footwear use [13,14]. It is also consistent with previous studies on other behaviors, such as the association of availability of fruit and vegetables at home with intake of these food items among children [52–53] and the association between access to sports facilities and physical activity among children and adolescents [22]. In the present study, ownership of footwear independently predicted the probability of using footwear regularly and optimally. Its interaction with family socioeconomic status on the protective use of footwear was also significant. This suggests that adequacy of footwear ownership among children is determined partly by the economic capacity of households. Thus, creating circumstances that increase the capacity of lower socioeconomic status families could play an important role in protective use of footwear.

### Limitations and future research directions

Though the findings presented in this study are of significance, the study is not without limitations. One of the limitations relates to measuring protective use of footwear. These days, researchers use sophisticated technologies to accurately measure routinized behaviors such as physical activity using pedometers, portable electronic or electromechanical devices that count every step a person takes in a day [54]. To date, there is no comparable way of measuring use of footwear. As a result, we relied on self-report over the last seven days, and observation on the day of interview. The self-report data is likely to be affected by recall bias. Daily diary records of observed use of footwear across situations within a certain range of days may be more accurate, but are also likely to be time intensive and costly. A previous study tried to check the veracity of self-reported use of footwear through observed foot condition (i.e. adherent soil, foot trauma, heel fissures and nail dystrophy) and sock/shoe imprints on foot [12]. However, the relationship between observed foot condition and reported shoe use was not clear. Future research efforts may focus on developing reliable and valid measures of footwear use. This is vital to understanding trends of footwear use as preventive health behavior and for determining the proportion of the rural population vulnerable to podoconiosis.

The low level of explained variance accounted for by the regression model consisting of family socioeconomic status, adequacy of footwear ownership and interaction term variables implies the importance of examining the role of other factors. In our previous qualitative study, misperception of risk for podoconiosis and perceived barriers to footwear use including uncomfortable footwear, shortage and poor adaptability of footwear for farm activities and sports were reported to negatively affect optimum use of footwear among school-age children [24]. Cultural, cognitive and interpersonal factors may interact with socioeconomic factors to influence protective footwear use in children. Future studies may therefore consider exploring the role these factors to further advance our understanding of the determinants of footwear use in the study area and elsewhere.

## Conclusion

Increased adoption of protective footwear is needed to effectively prevent school-age children living in endemic areas from developing podoconiosis and other neglected tropical diseases. Approaches aimed to encourage ownership of multiple pairs of footwear should also consider increasing the socio-economic capacity of families in podoconiosis endemic communities. Livelihood strengthening interventions that provide opportunities for skills development, self-employment, micro-credit and social protection services including educational support for children and access to free or subsidized health care may contribute to socio-economic empowerment of podoconiosis-affected families.

## Supporting information

S1 Text Survey Questionnaire. (DOCX)

## References

[1] Tomczyk S, Deribe K, Brooker SJ, Clark H, Knopp S, Utzinger J, et al. Association between Footwear Use and Neglected Tropical Diseases: A Systematic Review and Meta-Analysis. PLoS Negl Trop Dis. 2014;8(11): e3285. doi: 10.1371/journal.pntd.0003285 25393620 PMC4230915

[2] Hotez PJ., Molyneux DH, Fenwick A, Kumaresan J, Sachs SE, Sachs JD., et al. Control of neglected tropical diseases. N Engl J Med. 2007;357:1018–1027. doi: 10.1056/NEJMra064142 17804846

[3] WHO. 2010. Working to Overcome the Global Impact of Neglected Tropical Diseases: First WHO Report on Neglected Tropical Diseases. World Health Organization. http://whqlibdoc.who.int/publications/2010/9789241564090_eng.pdf.

[4] Davey G, GebreHanna E, Adeyemo A, Rotimi C, Newport M, Desta K. Podoconiosis: a Tropical Model for Gene-Environment Interactions? Trans R Soc Trop Med Hyg. 2007;101: 91–96. doi: 10.1016/j.trstmh.2006.05.002 16884751

[5] Tekola-Ayele F, Adeyemo A, Finan C, Hailu E, Sinnott P, Burlinson ND, et al. HLA Class II Locus and Susceptibility to Podoconiosis. N Engl J Med. 2012;366: 1200–1208. doi: 10.1056/NEJMoa1108448 22455414 PMC3350841

[6] Desta K, Ashine M, Davey G. Prevalence of Podoconiosis (Endemic Nonfilarial Elephantiasis) in Wolaitta, Southern Ethiopia. Trop Doct. 2003;33: 217–220. doi: 10.1177/004947550303300410 14620426

[7] Price EW, Henderson WJ. The Elemental Content of Lymphatic Tissues of Barefooted People in Ethiopia with Reference to Endemic Elephantiasis of the Lower Legs. Transactions of the Royal Society of Tropical Medicine and Hygiene. 1978;72:132–136. doi: 10.1016/0035-9203(78)90048-2 653784

[8] Davey G, Tekola F, Newport MJ. Podoconiosis: Non-Infectious Geochemical Elephantiasis. Trans R Soc Trop Med Hyg. 2007;101: 1175–1180. doi: 10.1016/j.trstmh.2007.08.013 17976670

[9] Ayode D, McBride CM., de Heer H, Watanabe E, Gebreyesus T, Tadele G, et al. The Association of Beliefs about Heredity with Preventive and Interpersonal Behaviors in Communities Affected by Podoconiosis in Rural Ethiopia. The American Journal of Tropical Medicine and Hygiene. 2012; 87(4): 623–630. doi: 10.4269/ajtmh.2012.12-0204 22826482 PMC3516310

[10] Tora A, Ayode D, Tadele G, Farrell D, Davey G, McBride CM. Interpretations of education about gene-environment influences on health in rural Ethiopia: the context of a neglected tropical disease. Int Health. 2016; doi: 10.1093/inthealth/ihw016 27114426 PMC4967847

[11] Ayode D, Tora A, Farrell D, Tadele G, Davey G, McBride CM. Association between Causal Beliefs and Shoe Wearing to Prevent Podoconiosis: A Baseline Study. Am. J. Trop. Med. Hyg. 2016;94(5): 1123± 1128 doi: 10.4269/ajtmh.15-0342 26928843 PMC4856613

[12] Emi W, McBride CM., Tora A, Ayode D, Farrell D, Davey G. Use of Footwear and Foot Condition among Rural Ethiopian School Children. J Epidemiol Global Health. 2014; 10.1016/j.jegh.2014.06.001 PMC7320331 25455650

[13] Ayode D, McBride CM, de Heer HD, Watanabe E, Gebreyesus T, Tora A, et al. A Qualitative Study Exploring Barriers Related to Use of Footwear in Rural Highland Ethiopia: Implications for Neglected Tropical Disease Control. *PLoS Negl Trop Dis* 2013;7(4): e2199. doi: 10.1371/journal.pntd.0002199 23638211 PMC3636134

[14] Kelemework A, Tora A, Amberbir T, Agedew G, Asmamaw A, Deribe K, et al. Why Should I Worry, Since I Have Healthy Feet?’ A Qualitative Study Exploring Barriers to Use of Footwear among Rural Community Members in Northern Ethiopia. BMJ Open. 2016; 6: e010354. doi: 10.1136/bmjopen-2015-010354 27006343 PMC4809094

[15] Cockerham WC. Health Lifestyles: Bringing Structure Back. In the New Blackwell Companion to Medical Sociology. Oxford: Blackwell Publishing Ltd. 2010. pp. 159–183

[16] Giddens A. The Constitution of Society: an Outline of the Theory of Structuration. Cambridge: Polity Press; 1984.

[17] Bandura A. Social cognitive theory: An agentic perspective. Asian Journal of Social Psychology. 1999;2: 21–41. 10.1146/annurev.psych.52.1.1 11148297

[18] Fernández-Ballesteros R, Díez-Nicolás J, Caprara GV, Barbaranelli C, Bandura A. Determinants and Structural Relation of Personal Efficacy to Collective Efficacy. Applied Psychology: An International Review. 2002;51 (1):107–125.

[19] Glymour MM, Avendano M, Kawachi I. Socioeconomic status and health. In *Social Epidemiology*. 2^nd^ ed. Oxford: Oxford University Press; 2014. pp 17–63

[20] Molla YB, Le Blond JS, Wardrop N, Baxter P, Atkinson PM, et al. Individual Correlates of Podoconiosis in Areas of Varying Endemicity: A CaseControl Study. PLoS Negl Trop Dis. 2013; 7(12): e2554. doi: 10.1371/journal.pntd.0002554 24340109 PMC3854961

[21] Tamiru A, Tsegay G, Wubie M, Gedefaw M, Tomczyk S, Tekola-Ayele F. Podoconiosis Patients’ Willingness to Pay for Treatment Services in Northwest Ethiopia: Potential for Cost Recovery. BMC Public Health 2014;14:259. doi: 10.1186/1471-2458-14-259 24642085 PMC4234032

[22] Sallis JF, Prochaska JJ, Taylor WC. A Review of Correlates of Physical Activity of Children and Adolescents. Med Sci Sports Exerc 2000;32: 963–75. doi: 10.1097/00005768-200005000-00014 10795788

[23] McMurray RG, Harrel JS, Bangdiwala SI, Hu J. Tracking of Physical Activity and Aerobic Power from Childhood Through Adolescence. Med Sci Sports Exerc. 2003;35(11):1914–22. doi: 10.1249/01.MSS.0000093612.59984.0E 14600559

[24] Tora A, Tadele G, Aseffa A, McBride CM, Davey G Health beliefs of school-age rural children in podoconiosis-affected families: A qualitative study in Southern Ethiopia. PLoS Negl Trop Dis. 2017;11(5): e0005564. doi: 10.1371/journal.pntd.0005564 28542227 PMC5444591

[25] Fleiss JL. Statistical Methods for Rates and Proportions. New York: John Wiley and Sons; 1981.

[26] Babbie E. The Practice of Social Research. Belmont: Wadsworth Cengage Learning; 2010.

[27] Deribe K, Cano J, Newport MJ, Golding N, Pullan RL, Sime H, et al. Mapping and Modelling the Geographical Distribution and Environmental Limits of Podoconiosis in Ethiopia. PLoS Negl Trop Dis. 2015;9(7): e0003946. doi: 10.1371/journal.pntd.0003946 26222887 PMC4519246

[28] Molla YB, Tomczyk S, Amberbir T, Tamiru A, Davey G. Patients’ Perceptions of Podoconiosis Causes, Prevention and Consequences in East and West Gojam, Northern Ethiopia. BMC Public Health. 2012;12:828. doi: 10.1186/1471-2458-12-828 23020758 PMC3519620

[29] Alemu G, Tekola-Ayele F, Daniel T, Ahrens C, Davey G. Burden of Podoconiosis in Poor Rural Communities in Gulliso woreda, West Ethiopia. PLoS NTD. 2011;5: e1184. doi: 10.1371/journal.pntd.0001184 21666795 PMC3110157

[30] Tekola-Ayele F, Alemu G, Davey G, Ahrens C. Community-based Survey of Podoconiosis in Bedele Zuria Woreda, West Ethiopia. Int Health 2013;5:119–125 doi: 10.1093/inthealth/iht003 24030111 PMC3889643

[31] Cockerham WC, Ritchey FJ. *Dictionary of Medical Sociology*. Westport: Greenwood Press; 1997.

[32] Ferdous HS. Asset-based poverty analysis in rural Bangladesh: A comparison of principal component analysis and fuzzy set theory. Sustainability Research Institute (SRI): University of Leeds; 2014.

[33] Filmer D, Pritchett LH. 2001. Estimating Wealth Effects without Expenditure Data-Or Tears: An Application to Educational Enrolments in States of India. Demography. 38(1):115–132. doi: 10.1353/dem.2001.0003 11227840

[34] Dekker M. Estimating Wealth Effects without Expenditure Data: Evidence from Rural Ethiopia. Ethiopian Journal of Economics. 2006; XV(1): 35–53.

[35] Kollmair M, Gamper St. “The Sustainable Livelihoods Approach.” Input Paper for the Integrated Training Course of NCCR North-South. Development Study Group, University of Zurich; 2002.

[36] Carswell G. Livelihood Diversification: Increasing in Importance or Increasingly recognized? Evidence from Southern Ethiopia. J. Int. Dev. 2002;14: 789–804.

[37] Eneyew A, Bekele W. Determinants of Livelihood Strategies in Wolaita, Southern Ethiopia. Agricultural Research and Reviews. 2012;1(5):153–161.

[38] Gecho Y, Ayele G, Lemma T, Alemu D. Rural Household Livelihood Strategies: Options and Determinants in the Case of Wolaita Zone, Southern Ethiopia. Social Sciences. 2014;3(3): 92–104. doi: 10.11648/j.ss.20140303.15

[39] Tekola F, H. Mariam D, Davey G. Economic Costs of Endemic Non-filarial Elephantiasis in Wolaita Zone, Ethiopia. Tropical Medicine and International Health. 2006;11(7):1136–1144. doi: 10.1111/j.1365-3156.2006.01658.x 16827714

[40] Tora A, Davey G, Tadele G. A Qualitative Study on Stigma and Coping Strategies of Patients with Podoconiosis in Wolaita Zone, Southern Ethiopia. Int Health. 2011;3: 176–181. doi: 10.1016/j.inhe.2011.06.006 24038367

[41] Tora A, Franklin H, Deribe K, Reda AA, Davey G. Extent of Podoconiosis-Related Stigma in Wolaita Zone, Southern Ethiopia: A Cross-Sectional Study.” SpringerPlus. 2014;3:647. doi: 10.1186/2193-1801-3-647 25485190 PMC4233027

[42] McBride CM, Price CS., Ayode D, Tora A, Farrell D, Davey G. A cluster randomized intervention trial to promote shoe use by children at high risk for podoconiosis. Int J Health Sci Res. 2015;5: 518–28.

[43] Evelyn D. Poverty and Gender Inequality in Developing Countries. Developing Country Studies. 2015;5(10):76–102.

[44] Bhalotra S, Rawlings SB. Intergenerational Persistence in Health in Developing Countries: The Penalty of Gender Inequality? Journal of Public Economics. 2011;95(3–4): 286–99.

[45] Jayachandran S. The Roots of Gender Inequality in Developing Countries. Northwestern University November. Prepared for Annual Review of Economics; 2014. http://faculty.wcas.northwestern.edu/~sjv340/roots_of_gender_inequality.pdf

[46] Clough S. Gender and the Hygiene Hypothesis. Soc Sci Med. 2011;72(4):486–93. doi: 10.1016/j.socscimed.2010.11.021 21195519

[47] Bandura A. Health Promotion from the Perspective of Social Cognitive Theory. Psychology and Health. 1998;13:623–649.

[48] Pampel FC, Krueger PM, Denney JT. “Socioeconomic Disparities in Health Behaviors.” Annu Rev Sociol. 2010;36: 349–370. doi: 10.1146/annurev.soc.012809.102529 21909182 PMC3169799

[49] Hanson MD, Chen E. “Socioeconomic Status and Health Behaviors in Adolescence: A Review of the Literature. J Behav Med. 2007;30:263–285 doi: 10.1007/s10865-007-9098-3 17514418

[50] Davison KK, Jurkowski JM, Lawson HA. Reframing family-centred obesity prevention using the Family Ecological Model. Public Health Nutr. 2013;16(10): 1861–1869. doi: 10.1017/S1368980012004533 23089267 PMC5500251

[51] Bere E, Klepp K-I. “Changes in Accessibility and Preferences Predict Children’s Future Fruit and Vegetable Intake.” The International Journal of Behavioral Nutrition and Physical Activity. 2005;2: 15–22. doi: 10.1186/1479-5868-2-15 16216124 PMC1262749

[52] Cullen KW, Baranowski T, Owens E, Marsh T, Rittenberry L, de Moor C. Availability, Accessibility, and Preferences for Fruit, 100% Fruit Juice, and Vegetable Influence Children’s Dietary Behavior. Health Education & Behavior. 2003;30: 615–626. 14582601 10.1177/1090198103257254

[53] Nepper MJ, Chai W. Associations of the Home Food Environment with Eating Behaviors and Weight Status among Children and Adolescents. J Nutr Food Sci. 2015. S12: 004. doi: 10.4172/2155-9600.S12-004

[54] Bravata D. Using Pedometers to Increase Physical Activity and Improve Health. The Journal of the American Medical Association. 2007;298 (19): 2296–304. doi: 10.1001/jama.298.19.2296 18029834

