# Supplementary material for: The extent of protective footwear use among school-age rural children at high risk for podoconiosis and socio-economic correlates: A household cross-sectional survey in Southern Ethiopia
Source: PLoS Negl Trop Dis. 2021 Oct 4;15(10):e0009791. doi: 10.1371/journal.pntd.0009791 (PMC8489712; doi:10.1371/journal.pntd.0009791)
Supplement: S1 Text — (DOCX) [file pntd.0009791.s001.docx]

**Supplementary information 1. Survey questionnaire**

**Family Members Enumeration Sheet**

**Woreda ………………………….**

To be attached with the questionnaire

**Kebele …………………..……….**

**Household code …………………**

**Household members’ enumeration sheet (those who live and eat together in the same house at the time of interview)**

| No. | Name | Age | Sex | Podoconiosis status | Relationship to members | Who is the household head?  (put X) | Randomly selected child for interview (put X) |
| --- | --- | --- | --- | --- | --- | --- | --- |
| 1 |  |  |  |  |  |  |  |
| 2 |  |  |  |  |  |  |  |
| 3 |  |  |  |  |  |  |  |
| 4 |  |  |  |  |  |  |  |
| 5 |  |  |  |  |  |  |  |
| 6 |  |  |  |  |  |  |  |
| 7 |  |  |  |  |  |  |  |
| 8 |  |  |  |  |  |  |  |
| 9 |  |  |  |  |  |  |  |
| 10 |  |  |  |  |  |  |  |
| 11 |  |  |  |  |  |  |  |
| 12 |  |  |  |  |  |  |  |

**Subject Information Sheet**

The purpose of this study is to examine the role of individual and socio-structural determinants of Footwear practice of rural children in podoconiosis affected families in southern Ethiopia. This is a study conducted by Mr Abebayehu Tora, a doctoral student in the department of sociology, Addis Ababa University. You are invited to take part in this study which aims to investigate determinants of footwear practice of rural children in podoconiosis affected families in Wolaita Zone, Southern Ethiopia. I seek your willingness to take part in this study, hoping that your contributions through providing genuine information will help me understand various forms of factors that influence the footwear practice of children at high risk to podoconiosis, and suggest feasible strategies of halting intergenerational continuity of podoconiosis in your community. The findings of this study will be used to inform policy making and implementation that aim to ensure the accessibility and effectiveness of tailored prevention and control programs in podoconiosis endemic communities in Ethiopia and elsewhere. The study targets families affected by podoconiosis. Both parent household heads and their children between the age of 9-15 years will be interviewed based on the questionnaires and interview guides. The study will be conducted in three districts of Wolaita Zone.

I would like to ask if you are willing to participate in this study. You may devote a maximum of 1hr to the study. Your participation in the study is based completely on your voluntariness. I would like to emphasize that you have the right to stop being part of the research at any time without explanation. You have the right to ask that any data you have supplied to that point be erased. You have the right to omit or refuse to answer or respond to any question that is asked of you. Interviews will be conducted individually in a private location. Once the data have been collected, you will be de-identified, your information will be assigned a study ID number and your name will not be linked to this number. All your information will be stored in a protected file. If you have any questions as a result of reading this information sheet, I, Abebayehu Tora, will be glad to answer your questions about this study at any time. You may contact me at mobile number 0913190253.

Thank you in advance for considering taking part in this study!

**Consent Sheet for household heads**

My Name is ……………………………….. I am one of the data collectors for the study entitled ***“Footwear practice of rural children at high risk for podoconiosis in Southern Ethiopia: examining the joint role of individual and socio-structural determinants”.*** The study involves podoconiosis affected families in Wolaita zone. Your family has been selected for this study based on the information we obtained from the Mossy Foot International [a non-governmental organization that provides treatment to podoconiosis patients in Wolaita Zone] that one of your family member has been receiving podoconiosis treatment in the last few years. As it has been indicated in household members’ enumeration sheet, you are a head of this household. Today, I came to your house to collect information on how you perceive the causes and prevention mechanisms of podoconiosis, and what costs and benefits you associate with footwear practice. I would like to interview you and your child (mention the name of the child)……………………………….based on a list of questions. I will continue interviewing you and your child only if you are willing to participate or let your child participate in the study. You have full right to avoid participation in the study. And, if you feel discomfort during interviews, you have the right to withdraw from interviews at any stage. The information you give me will be recorded anonymously, will not be transferred to the third party and will be utilized only for the research purposes. I have a list of questions on which I expect your answers. I will also be asking your child more or less similar questions. I will read the questions after clearly indicating how you will respond. Feel free to give only the answers that you think right and genuine. I am here to learn from you. This is not like a classroom examination. Any of your answers are very important to our study. The interview may last about 1hour.

Finally, if you have any questions please ask either during the interview or afterwards.

The study has been explained to me and I agree to participate. I acknowledge that I can withdraw from the study at any time without any consequences.

Signature/thumb print/ of the participant …………………………….

I also agree to give permission that my son/daughter (child name) ……………………… can participate in the study. I further acknowledge that she/he can withdraw from the study any time without any consequences.

Date: ……………………………

Signature/thumb print/ of the participant …………………………….

- [If the household head is not willing to participate, please thank him for his/her time and leave the house].
- [If the household head is willing to participate while being not willing to give permission for his/her child, please thank him for his willingness but don’t continue interviewing as both parents and their children should take part]
- [If the household head is willing to participate and gives permission to his/her child as well, please thank him or her in advance for his or her willingness and start interviewing].

**Assent Sheet for Children**

My Name is ……………………………….. I am one of the data collectors to the study entitled ***“Footwear practice of rural children at high risk for podoconiosis in Southern Ethiopia: examining the joint role of individual and socio-structural determinants”.*** The study involves 9-15 years old rural children and their parents as well. We selected you for this study randomly from other children in your family within the age of 9-15 years. Today, I came to your house to collect information on how you perceive the causes and prevention mechanisms of podoconiosis, and what costs and benefits you associate with footwear practice. Your parents gave me permission to approach you for interview. I will continue interviewing you only if you are willing to participate. You have full right to avoid participation in the study. And, if you feel discomfort during interviews, you have the right to withdraw from interviews at any stage. The information you give me will be recorded anonymously, will not be transferred to the third party including your parents or siblings, and will be utilized only for the research purposes. I have a list of questions on which I expect your answers. I will read the questions after clearly indicating how you will respond. Feel free to give only the answers that you think right and genuine. I am here to learn from you. This is not like a classroom examination. Whatever you say is very important to me. The interview may take about 45 minutes.

Finally, if you have any questions please ask either during the interview or afterwards.

The study has been explained to me and I agree to participate. I acknowledge that I can withdraw from the study at any time without any consequences.

Date: ……………………………

Signature/thumb print …………………………….

[If the child is not willing to participate, please thank him for his/her time and leave the house].

[If the child is willing to participate, please thank him or her in advance for his or her willingness and start interviewing].

1. **Socio-demographic information of children**
2. How old are you? ……………
3. Sex: Male ………… 1

Female ……… 2

1. Have you attended school?

No ………. 0

Yes ……… 1

1. If you attended school, what is the highest grade level you attained so far?

**Grade** ………

1. **Adequacy of footwear ownership index for children**

| No. | Question | Response | | |
| --- | --- | --- | --- | --- |
|  |  |  |  |  |
| 1 | Do you have shoes of any sort? | No ……………….. 0  Yes ………………. 1 | | |
| If Yes, how many pairs of shoes do you have? | | None | 1 pair | ≥2 pairs |
| 1 | Open plastic (foam) | 0 | 1 | 2 |
| 2 | Open leather | 0 | 1 | 2 |
| 3 | Closed plastic (foam) | 0 | 1 | 2 |
| 4 | Closed leather | 0 | 1 | 2 |
| 5 | Closed canvass (sneaker) | 0 | 1 | 2 |

1. **Protective footwear use index for children**

| No. | | Question | Response | | |
| --- | --- | --- | --- | --- | --- |
| In the last 7 days, which type of shoes did you wear for the following activities? | | | None | Open  shoes | Closed shoes |
| 1 | When performing domestic chores | | 0 | 1 | 2 |
| 2 | When performing farming activities | | 0 | 1 | 2 |
| 3 | While cutting grass | | 0 | 1 | 2 |
| 4 | When collecting fuel wood | | 0 | 1 | 2 |
| 5 | When looking after cattle | | 0 | 1 | 2 |
| 6 | When fetching water | | 0 | 1 | 2 |
| 7 | When playing games at school | | 0 | 1 | 2 |
| 8 | When playing games after school | | 0 | 1 | 2 |
| 9 | Walking around homestead (as observed at the time of interview) | | 0 | 1 | 2 |
| 10 | When going to nearby market in the village | | 0 | 1 | 2 |
| 11 | When going to big market in the town | | 0 | 1 | 2 |
| 12 | When going to school | | 0 | 1 | 2 |
| 13 | When going to church | | 0 | 1 | 2 |

1. **Family socioeconomic status index for household heads (Please put 0 in the blank space if the response is “none”)**

| **No.** | **Indicators of assets** | | **Response** | |
| --- | --- | --- | --- | --- |
|  |  |  | No (0) | Yes (1) |
|  | **Human capital** | |  |  |
| 1 | Household size is greater than 5 | |  |  |
| 2 | Family members are within 15-65 years of age | |  |  |
| 3 | There are family members completing primary education | |  |  |
| 4 | There are family members completing secondary education | |  |  |
| 5 | There are family members with college diploma | |  |  |
| 6 | Household head is podoconiosis affected | |  |  |
| 7 | There are household members with college degree | |  |  |
| 8 | There are household members with hand craft skills | |  |  |
|  | **Social capital** | |  |  |
| 9 | Family members have Equb membership | |  |  |
| 10 | Household members have *edir* membership | |  |  |
| 11 | Family members have membership in saving and credit  Association | |  |  |
| 12 | Family members have membership in agricultural  Cooperatives | |  |  |
| 13 | Family members have membership in local cooperative farming systems | |  |  |
| 14 | Family members have membership in community leadership positions | |  |  |
|  | **Financial capital** | |  |  |
| 15 | Money deposited in Equb | |  |  |
| 16 | Money deposited in saving and credit association | |  |  |
| 17 | Money deposited in Tekemach | |  |  |
| 18 | Ownership of horse or mule | |  |  |
| 19 | Oxen | |  |  |
| 20 | Cows | |  |  |
| 21 | Bulls | |  |  |
| 22 | Heifers | |  |  |
| 23 | Lamb | |  |  |
| 24 | Donkey | |  |  |
| 25 | Sheep | |  |  |
| 26 | Goats | |  |  |
| 27 | Chicken | |  |  |
| 28 | There are family members earning money from wage labor | |  |  |
| 29 | There are family members earning money from pity trade | |  |  |
| 30 | There are family members earning money from safety net program | |  |  |
| 31 | Household members received pension in the last six months | |  |  |
| 32 | Received money through charity in the last six months | |  |  |
| 33 | There are family members earning permanent salary | |  |  |
| 34 | Household members earned money from handcraft activity in the last six months | |  |  |
| 35 | Household members received remittance in the last six months | |  |  |
|  | **Physical capital** | |  |  |
| 36 | Tape recorder/radio | |  |  |
| 37 | Cell of phone | |  |  |
| 38 | Sponge mattress | |  |  |
| 39 | Wooden family bed | |  |  |
| 40 | Separate kitchen | |  |  |
| 41 | Armchair | |  |  |
| 42 | Roof covering | Grass |  |  |
|  |  | Iron sheet |  |  |
| 43 | Perceived condition of dwelling unit | Good |  |  |
|  |  | Bad |  |  |
| 44 | Perceived size of dwelling unit | Large |  |  |
|  |  | Small |  |  |
| 45 | Household members own bicycle | |  |  |
| 46 | Household members own motor cycle | |  |  |
| 47 | Household members own horse carriage | |  |  |
| 48 | Household members own donkey carriage | |  |  |
| 49 | Plastered walls | |  |  |
| 50 | Painted walls | |  |  |
|  | **Natural capital** | |  |  |
| 51 | Ownership of agricultural land | |  |  |
